# A C4 orphan crop, *Gynandropsis gynandra*, joins the genome club

**DOI:** 10.1093/plcell/koad043

**Published:** 2023-02-16

**Authors:** Kutubuddin A Molla

**Affiliations:** Assistant Features Editor, The Plant Cell, American Society of Plant Biologists, USA; Crop Improvement Division, ICAR-National Rice Research Institute, Cuttack 753006, India

Only 30 plant species fulfill 95% of human calorie needs, even though ∼300 species have been domesticated. As the human population crossed the 8 billion mark, the diversification of crop production and the inclusion of orphan crops into mainstream agriculture is necessary for the world's food security. Orphan crops are not traded internationally but are major food sources for millions of people, especially in parts of Africa, Asia, and South America. The development of genomic resources of orphan crops opens up new avenues to utilize them for modern crop breeding and sustainable food systems.

In this issue of *The Plant Cell*, **Nam V. Hoang and colleagues** (Hoang et al. 2023) present a chromosomal genome assembly for *Gynandropsis gynandra,* a C4 species considered an orphan crop in the spider flower family (Cleomaceae, Brassicales). *G. gynandra* is a leafy vegetable and medicinal plant found across the world, and it has been used as a model to study C4 photosynthesis. The authors constructed a high-quality genome assembly of *G. gynandra* using a combined method of Illumina sequencing, 10× Genomics sequencing, and chromatin conformation capture (Hi-C) technologies.

In an effort to unravel the evolutionary history of C4 photosynthesis in *Gynandropsis,*Hoang et al. (2023) compared their genome data with data available for a closely related C3 species, *Tarenaya hassleriana* (Cheng et al. 2013). Comparative analyses revealed that a whole-genome duplication in the common ancestor of the two species, followed by differing patterns of loss and retention, contributed to the expansion of gene family size for C4-associated genes in *G. gynandra*. *T. hassleriana* gains a third genome copy following divergence from the lineage leading to *G. gynandra* (see Figure). Nonetheless, the *G. gynandra* genome retained two or more copies of 29 genes involved in the NAD-ME subtype of C4-related biochemical reactions, whereas *T. hassleriana* retained significantly fewer gene copies. The elevated numbers of C4-associated genes in *G. gynandra* were derived from both polyploidy and single-gene duplications. Taken together, differential duplication, biased retention, recruitment, and expression modification of the C4-related gene families facilitate the evolution of the C4 cycle in *G. gynandra*.

**Figure.**
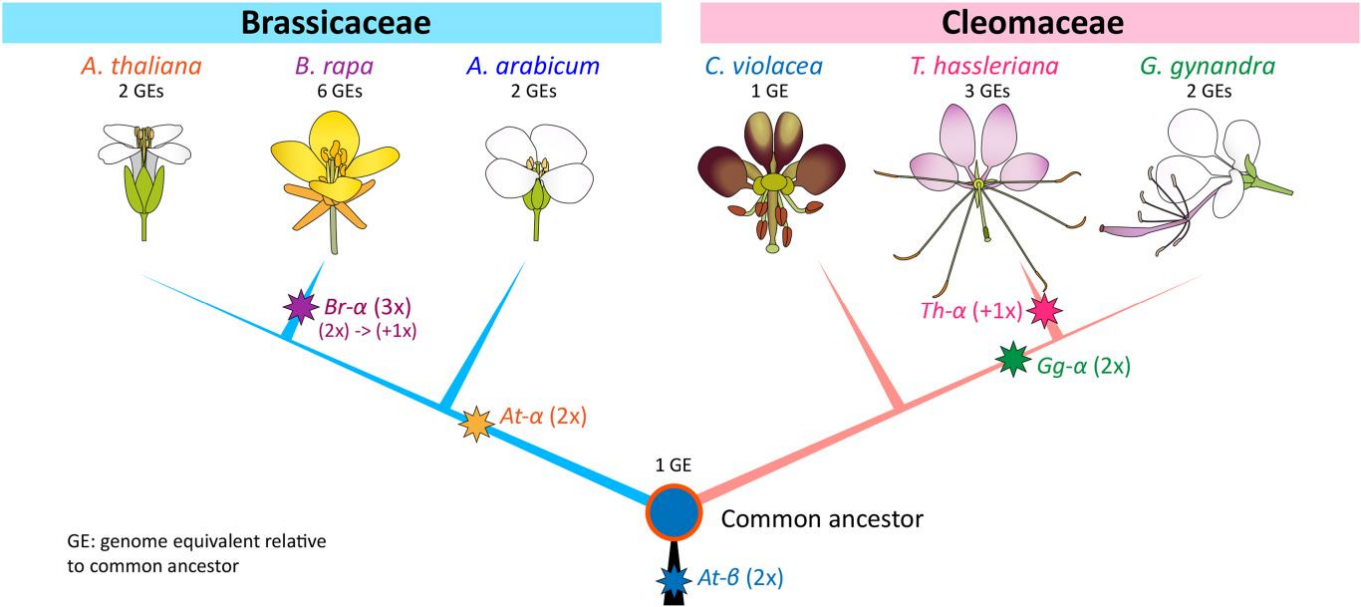
Ancient polyploidy events and phylogenetic relationship between Brassicaceae and Cleomaceae genera/species. Adapted from Hoang et al. (2023), Figure 3F.

The assembled *G. gynandra* genome size is ∼740 Mb with 30,933 annotated gene models. Approximately 30,000 genes (97% of the total) were supported by transcriptome data from 18 different tissues previously reported by Külahoglu et al. (2014). About 99% of the assembled genome was anchored onto 17 super-scaffolds (pseudo-molecules), in agreement with an earlier estimation of chromosome numbers (2*n* = 34) for *G. gynandra* (Omondi et al. 2017). Repetitive elements, including long terminal repeat retrotransposons and DNA transposons, accounted for 69% of the assembled genome.

Genomic resources of phylogenetic outgroups are essential for inferring evolutionary trajectories. Cleomaceae is a sister family of Brassicaceae to which the model plant *Arabidopsis* (a C3 species) belongs. The Cleomaceae contains species with different types of photosynthesis, including C3, C4, and C3–C4 intermediate plants, whereas there are no true C4 species in the Brassicaceae. With the high-quality genome from Hoang et al., *G. gynandra* can be used as a suitable C4 model species opening new avenues for comparative functional and evolutionary studies of C3 and C4 photosynthesis. Additionally, the genome information of this orphan crop is a valuable resource for its improvement and future inclusion into mainstream agriculture.
